# Alternative Splicing Diversified the Heat Response and Evolutionary Strategy of Conserved *Heat Shock Protein 90s* in Hexaploid Wheat (*Triticum aestivum* L.)

**DOI:** 10.3389/fgene.2020.577897

**Published:** 2020-11-27

**Authors:** Yunze Lu, Peng Zhao, Aihua Zhang, Lingjian Ma, Shengbao Xu, Xiaoming Wang

**Affiliations:** ^1^State Key Laboratory of Crop Stress Biology for Arid Areas, College of Agronomy, Northwest A&F University, Yangling, China; ^2^School of Landscape and Ecological Engineering, Hebei University of Engineering, Handan, China

**Keywords:** wheat, heat stress, HSP90, alternative splicing, evolutionary conservation and divergence

## Abstract

Crops are challenged by the increasing high temperature. Heat shock protein 90 (HSP90), a molecular chaperone, plays a critical role in the heat response in plants. However, the evolutionary conservation and divergence of *HSP90s* homeologs in polyploidy crops are largely unknown. Using the newly released hexaploid wheat reference sequence, we identified 18 *TaHSP90s* that are evenly distributed as homeologous genes among three wheat subgenomes, and were highly conserved in terms of sequence identity and gene structure among homeologs. Intensive time-course transcriptomes showed uniform expression and transcriptional response profiles among the three *TaHSP90* homeologs. Based on the comprehensive isoforms generated by combining full-length single-molecule sequencing and Illumina short read sequencing, 126 isoforms, including 90 newly identified isoforms of *TaHSP90s*, were identified, and each *TaHSP90* generated one to three major isoforms. Intriguingly, the numbers and the splicing modes of the major isoforms generated by three *TaHSP90* homeologs were obviously different. Furthermore, the quantified expression profiles of the major isoforms generated by three *TaHSP90* homeologs are also distinctly varied, exhibiting differential alternative splicing (AS) responses of homeologs. Our results showed that the AS diversified the heat response of the conserved *TaHSP90s* and provided a new perspective for understanding about functional conservation and divergence of homologous genes.

## Introduction

As a result of global warming and increasing frequent short episodes of extreme high temperature (Liu et al., 2014; Prasad and Djanaguiraman, 2014), heat stress has become one of the major factors that limit crop production and quality (Asseng et al., 2015; Lesk et al., 2016; Zhao et al., 2017). Each degree Celsius increase in global mean temperature would reduce crop yield by 6–10% under heat stress (Lesk et al., 2016; Zhao et al., 2017).

Plants have evolved complex systems to cope with heat stress. Heat shock proteins (HSPs), which function as molecular chaperones, have well-established roles in the heat response (Wang et al., 2004). Ninety kDa HSPs (HSP90s) function not only in protein folding, degradation, and transportation, as characteristic of chaperones, but also in signaling transduction and protein kinase activity regulation, in an ATP-dependent manner (Carretero-Paulet et al., 2013; Schopf et al., 2017). Under heat stress, the 70-kDa HSP/HSP90 complex disassociates and releases the master heat response regulator, heat shock transcription factor A1s (HSFA1s), resulting in the activation of the plant heat-responsive transcription cascade (Hahn et al., 2011; Ohama et al., 2017). More recently, HSP90s have been proven to have the ability to stabilize the circadian clock ZEITLUPE and auxin cofactor F-box protein to maintain plant growth under heat stress (Wang et al., 2016; Gil et al., 2017). Under normal conditions, the downregulated expression of Arabidopsis HSP90s results in abnormal growth and development, such as embryo defects (Queitsch et al., 2002). HSP90s are also considered to exert capacitor-buffering effects of genetic perturbation, such as genetic variations and mutations, on plant morphology and phenotype (Rutherford and Lindquist, 1998; Geiler-Samerotte et al., 2016).

The identification and expression pattern analysis of *HSP90*s have been widely reported in Arabidopsis, rice, chickpea, and pigeonpea (Krishna and Gloor, 2001; Swindell et al., 2007; Hu et al., 2009; Agarwal et al., 2016). In Arabidopsis, seven *AtHSP90s* were characterized and classified into different subfamilies based on their subcellular localization. Under normal condition, *HSP90AA* (*AtHSP90-1*) and *HSP90ABs* (*AtHSP90-2*-*AtHSP90-4*) are located in the cytoplasm, and *HSP90C1* (*AtHSP90-5*), *HSP90C2* (*AtHSP90-6*), and *HSP90B* (*AtHSP90-7*) are located in chloroplasts, mitochondria, and endoplasmic reticulum, respectively. Under heat stress, HSP90s were found to translocate into the nucleus, to regulate the expression of heat stress-responsive genes (Meiri and Breiman, 2009; Wang et al., 2016). The *HSP90AAs* are highly heat inducible, whereas *HSP90ABs* are constitutively expressed in Arabidopsis and rice (Yabe et al., 1994; Swindell et al., 2007; Hu et al., 2009). Similar heat response patterns of *HSP90s* within a subfamily are observed in *Populus* and allotetraploid tobacco (Zhang et al., 2013; Song et al., 2019). Meanwhile, previous studies also demonstrate the conservation in terms of exon–intron structures and protein motifs of *HSP90s* within a subfamily during plant evolution (Chen et al., 2006; Xu et al., 2012; Zhang et al., 2017).

Hexaploid wheat (*Triticum aestivum* L.), a major crop worldwide, contains three subgenomes, AA, BB, DD. These three subgenomes derive from three diploids through two major hybridization events. The first hybridization occurs between the diploid *T. urartu* (AA progenitor) and an unknown diploid Aegilops species (BB progenitor), possibly *Aegilops sharonensis* or *Ae. speltoides*, leading to the emergence of allotetraploid wild emmer wheat (*T. turgidum* ssp. *Dicoccoides*, AABB). The wild emmer wheat subsequently evolves into durum wheat (*T. turgidum* ssp. *Durum*, AABB). The second hybridization between tetraploid emmer wheat and the diploid *Ae. tauschii* (DD progenitor) results in the hexaploid wheat (AABBDD) (Dubcovsky and Dvorak, 2007; Marcussen et al., 2014). Due to the short polyploidy history, the hexaploid wheat is used as a model in the field of polyploidy evolution study, and the functional conservation and divergence of the homeologous genes in hexaploid wheat have always been intriguing (Borrill et al., 2015; Ramírez-González et al., 2018). At transcriptional level, expression partitioning of wheat homeologous genes is commonly observed, and the ratio of partially expressed genes ranges from about 55% in normal condition to around 68% in stress condition such as heat, drought, and salt (Leach et al., 2014; Liu et al., 2015; Zhang et al., 2016). Thus, expression partitioning of homeologous genes is thought to be a common strategy for abiotic stress acclimation in hexaploid wheat (Liu et al., 2015; Zhang et al., 2016).

In hexaploid wheat, nine cytosolic *TaHSP90*s have been reported, of which three *TaHSP90AA*s are highly expressed in the reproductive organs that are necessary for seedling growth, while six *TaHSP90AB*s are constitutively expressed that are essential for disease resistance (Wang et al., 2011). However, the evolution process of *HSP90s* during wheat polyploidization and the functional conversation and divergence between *TaHSP90* homeologs in hexaploid wheat remain largely limited.

With posttranscriptional regulation, many heat response genes, including several types of transcription factors and *HSPs*, change their alternative splicing (AS) patterns and generate new isoforms, expanding the diversity of proteome and regulation modes in the heat response (Jiang et al., 2017; Keller et al., 2017; Liu et al., 2018). For example, under normal conditions, *HSFA2* mainly encodes a truncated isoform without transcriptional activation activity, while under heat stress, the intact and transcriptionally active isoform is largely expressed to induce the expression of heat response genes (Sugio et al., 2009; Cheng et al., 2015). Interestingly, a new, small truncated form is induced, and this results in the autoregulation of *HSFA2* under severe heat stress (Liu et al., 2013). Whether and how *HSP90*s respond to heat stress with posttranscriptional regulation remains to be determined. If it is, what posttranscriptional regulation means for *HSP90s* is intriguing. The advantages of full-length single-molecule sequencing with long sequencing read provide powerful tool for accurate isoform detection and could be suitable to answer this question.

In this study, we performed a genome-wide identification of the *TaHSP90s* in hexaploid wheat using the newly released wheat reference sequence (IWGSC RefSeq v1.0) (International Wheat Genome Sequencing Consortium [IWGSC], 2018) and investigated the transcriptional and AS reprogramming of *TaHSP90s* using the dynamic and intensive heat response transcriptomes generated by combining full-length single-molecule sequencing and Illumina short read sequencing in our previous study (Wang et al., 2019). The 18 *TaHSP90s* were highly conserved in terms of sequence and transcriptional response pattern among *TaHSP90* homeologs, while the number, the splicing modes, and the AS responses of the major isoforms generated by *TaHSP90s* homeologs were distinctly different. Our findings indicated that AS regulation diversified the heat response of the conserved *TaHSP90* homeologs and possibly facilitated the evolutionary divergence of *TaHSP90* homeologs.

## Materials and Methods

### Identification of HSP90s in Hexaploid Wheat and Its Progenitors

Genome and protein sequences and genome annotation files of hexaploid wheat were downloaded from URGI^1^, and the protein sequences of *Ae. tauschii*, *T. urartu*, durum wheat, and wild emmer wheat were downloaded from the EnsemblPlants database^2^. Genome and protein sequences of *Ae. sharonensis* and *Ae. speltoides* were obtained from PGSB PlantsDB^3^. A blastp search was performed against the protein sequences of the above species using the HSP90 sequences of Arabidopsis and rice as queries with the following parameters: an e-value lower than 1e-5 and an identity score above 50%. Using the HMM profiles of the HSP90 domain (PF00183) downloaded from the Pfam database, the Hmmsearch engine in the HMMER3.0 program was also used to search these proteins with a threshold of 1e-5. Then, the blastp and HMMER results were merged, and redundancy was removed. All of the obtained HSP90 candidate sequences were subjected to search against the SMART database^4^ (Letunic and Bork, 2018) to manually confirm the presence of the histidine kinase-like ATPases (HATPase_c) and HSP90 domains (Zhang et al., 2013; Agarwal et al., 2016). Candidates that contained both the HATPase_c and HSP90 domain were regarded as HSP90s.

### Phylogenetic Relationship Analysis

Multiple sequence alignment was performed by MAFFT (L-INS-I algorithm) program using the protein sequences. Sequences from Arabidopsis and rice were used as markers to clarify the phylogeny. To investigate the evolutionary relationship of HSP90, a maximum likelihood tree was constructed using IQ-TREE (Nguyen et al., 2015). The substitution model was calculated with ModelFinder (integrated in IQ-TREE; best-fit model: JTT + G4 chosen according to the Bayesian information criterion) (Kalyaanamoorthy et al., 2017). The phylogenetic tree was examined by Ultrafast bootstraps (with parameters “-bb 1,000 -bnni”) as well as a Shimodaira–Hasegawa approximate likelihood ratio test (SH-aLRT, with parameters “-alrt 1,000”) (Guindon et al., 2010; Minh et al., 2013; Hoang et al., 2018). The tree file was visualized by Interactive Tree Of Life (iTOL) v4^5^ (Letunic and Bork, 2019).

### Naming of Hexaploid Wheat *HSP90s*

For clarity, we renamed all the *HSP90s* of hexaploid wheat, taking into account the naming convention of *HSP90* family members in Arabidopsis and rice. Each gene name started with the abbreviation of the hexaploid wheat (*T. aestivum*, *Ta*), followed by the abbreviation of this gene family (*HSP90*) and the subfamily name that is in Arabidopsis (AA, AB, B, C1, and C2), and finally ended with the chromosome and subgenome information. For example, the name of gene with geneID “*TraesCS2A01G033700*” was renamed as *TaHSP90AA-2A*; in other words, it was a *HSP90* gene belonging to the subfamily AA, and it was located on the AA subgenome on chromosome group 2. *HSP90s* from progenitors of hexaploid wheat were only presented in the phylogenetic relationship analysis; thus, they were not renamed.

### Transcriptional Regulation and AS Analysis of *TaHSP90s*

The transcriptome data were obtained from our previous research (Wang et al., 2019). Briefly, hexaploid wheat plants (*T. aestivum* cv. Chinese Spring) were grown in greenhouse under normal condition. After 15 days from anthesis, the plants were subjected to heat stress (37/17°C, 14/10 h). The filling grains and flag leaves were sampled at different time points and then for RNA isolation. The RNA-seq data, generated by Illumina (150 bp paired-end sequencing by Illumina’s HiSeq X Ten platform) and PacBio (PacBio RS II platform) sequencing, and data processing, isoform characterization were performed as described in a previous study (Wang et al., 2019). Briefly, the PacBio sequencing full-length and non-chimeric (FLNC) reads were first mapped to the bread wheat reference genome, and the mapped FLNC reads were filtered and corrected with our previously defined criterion. Then, the FLNC reads that were mapped to the same genome loci and shared the same splicing junctions were collapsed into one isoform. Finally, the identified isoforms were further filtered based on the number of FLNC reads supporting this isoform, percentage-of-identity of FLNC read-genome alignments, and the junction site verifications, whether they were supported by Illumina reads or genomic annotations as our previous description. The abundances of genes and isoforms were calculated by fragments per kilobase of exon per million fragment mapped (FPKM) values. The heat maps were drawn by “pheatmap” package in R software (version 3.6.1) with log2-transformed (FPKM + 1) values (Kolde and Kolde, 2015). Differentially expressed genes were identified by EdgeR (Robinson et al., 2010), and the genes that displayed fold change ≥ 2 and an FDR adjusted *P*-value < 0.01 were defined as differentially expressed genes, with non-stressed samples as the control. The gene with either of the following situation was regarded as a differentially spliced gene: (i) isoform set (FPKM ≥ 1 and fully supported by Illumina reads) differed between heat stress and control sample. (ii) The isoform expression percentage (IEP) changed by more than 30% between the control and heat stress samples (Wang et al., 2019).

## Results

### *HSP90* Genes in Hexaploid Wheat and Its Progenitors

**FIGURE 1:**
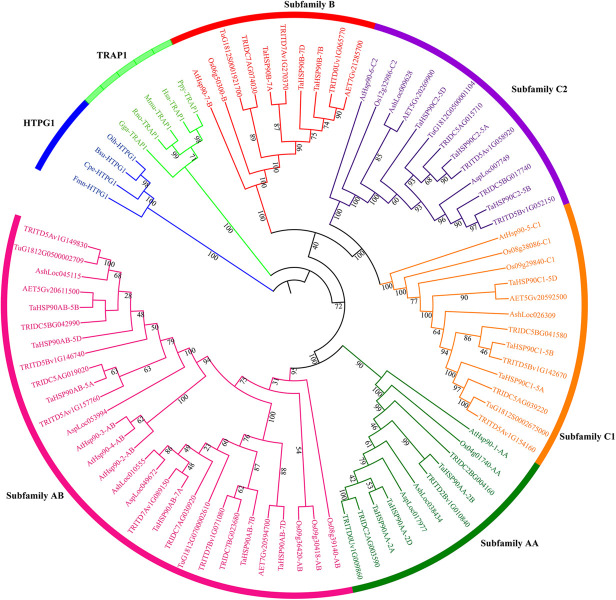
Maximum likelihood phylogeny of HSP90 proteins. HSP90 sequences were from Arabidopsis, rice, *T. urartu* (Tu), *Ae. sharonensis* (Ash), *Ae. speltoides* (Asp), *Ae. tauschii* (Aet), wild emmer wheat (*T. turgidum* ssp. *dicoccoides*, TRIDC), durum wheat (*T. turgidum* ssp. *durum*, TRITD), and hexaploid wheat (*T. aestivum*, Ta). The phylogenetic tree was inferred by IQ-TREE with Ultrafast bootstraps (1,000 replicates) and visualized by the Interactive Tree Of Life (Itol). Subfamilies were classified according to Arabidopsis and rice HSP90 subfamilies and showed by different colors. Bootstrap values were shown near the branches. HTPG1 (high-temperature protein G 1), the HSP90 homeolog in Eubacteria, and TRAP1 (tumor necrosis factor receptor-associated protein 1), which was the mitochondrial HSP90 homeolog in Animalia, were set as outgroups according to Chen et al. (2006). Bsu, *Bacillus subtilis*; Cpe, *Clostridium perfringens*; Fmn, *Fusobacterium nucleatum*; Gga, *Gallus gallus*; Hsa, *Homo sapiens*; Mmu, *Mus musculus*; Oih, *Oceanobacillus iheyensis*; Ppy, *Pongo pygmaeus*; Rno, *Rattus norvegicus*.

As a result of sequence searches and domain confirmation, 5, 5, 4, 5, 11, 13, and 18 *HSP90s* were identified in *T. urartu* (AA progenitor), *Ae. sharonensis* (possible BB progenitor), *Ae. speltoides* (possible BB progenitor), *Ae. tauschii* (DD progenitor), wild emmer wheat, durum wheat, and hexaploid wheat genome, respectively. According to the phylogenetic tree, these *HSP90s* were clearly classified into subfamily AA, AB, B, C1, and C2 referred to as the classification of Arabidopsis and rice (Figure 1).

**FIGURE 2:**
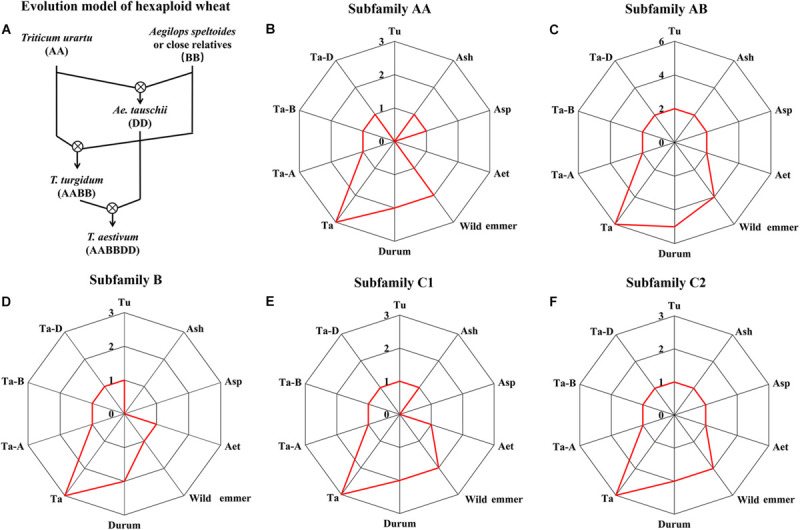
Copy number of each HSP90 subfamily in seven Triticeae species. **(A)** Evolution model of hexaploid wheat. The symbol “⊗” indicated a hybridization event. The copy number of HSP90s in different species is shown as red lines in **(B)** for subfamily AA, **(C)** for subfamily AB, **(D)** for subfamily B, **(E)** for subfamily C1, and **(F)** for subfamily C2. Abbreviations: Tu, *T. urartu*; Ash, *Ae. sharonensis*; Asp, *Ae. speltoides*; Aet, *Ae. tauschii*; Wild emmer, *T. turgidum* ssp. *dicoccoides*; Durum, *T. turgidum ssp. durum*; Ta, *T. aestivum*. Ta-A, -B, -D indicated subgenome AA, BB, and DD of hexaploid wheat, respectively.

The clear polyploidization process of hexaploid wheat provided an opportunity to study the evolution of *HSP90s* during this process (Figure 2A) (Dubcovsky and Dvorak, 2007; Marcussen et al., 2014). The numbers of *HSP90s* were around five for the diploid species, and the number in allotetraploid wild emmer wheat (11) seemed to be the sum of the two diploid progenitors. However, the number of *HSP90s* in hexaploid wheat (18) was somehow not the sum of the allotetraploid wild emmer wheat (11) and the diploid *Ae. tauschii* (5) (Figure 2). For example, the hexaploid and the allotetraploid wheat contain three and two *HSP90AA* members, respectively, but the *HSP90AA* members were absent in diploid progenitor *T. urartu* and *Ae. tauschii* genomes. Syntenic analysis showed that the genomic segment containing the *HSP90AA* members and other three neighborhood genes were absent in the two diploid genomes (Supplementary Figure 1), suggesting hexaploid, and the allotetraploid wheat may acquire the gene copy during polyploidization, or the diploid genomes were not completely assembled. Conclusively, the copy number variation of *HSP90* was not completely consistent with the polyploidy level.

### *TaHSP90s* Are Highly Conserved Between Three Homeologs

The 18 identified *HSP90s* in hexaploid wheat (TaHSP90s) were present in homeologous chromosome groups 2, 5, and 7 and were classified into *TaHSP90AAs* (3), *TaHSP90ABs* (6), *TaHSP90Bs* (3), *TaHSP90C1s* (3), and *TaHSP90C2s* (3) based on the phylogenetic tree (Figure 1 and Table 1). Intriguingly, the *TaHSP90s* in each subfamily were evenly distributed among the AA, BB, and DD subgenomes, and the three *TaHSP90s* on the same chromosome group in each subfamily were regarded as the three *TaHSP90* homeologs here after.

**TABLE 1:** Summary information of *TaHSP90s*.

***TaHSP90s***	**GeneID**	**Exon number**	**Isoform number**	**FPKM values**	
				**Grains**	**Flag leaves**
*TaHSP90AA-2A*	*TraesCS2A01G033700*	3	5	22–363	2–1,896
*TaHSP90AA-2B*	*TraesCS2B01G047400*	3	6	9–275	2–1,486
*TaHSP90AA-2D*	*TraesCS2D01G033200*	3	8	50–541	18–2,496
*TaHSP90AB-7A*	*TraesCS7A01G242200*	3	10	61–445	148–2,249
*TaHSP90AB-7B*	*TraesCS7B01G149200*	3	13	163–555	160–2,045
*TaHSP90AB-7D*	*TraesCS7D01G241100*	3	6	83–378	94–1,361
*TaHSP90AB-5A*	*TraesCS5A01G260600*	3	6	159–241	141–350
*TaHSP90AB-5B*	*TraesCS5B01G258900*	3	9	79–181	88–589
*TaHSP90AB-5D*	*TraesCS5D01G268000*	3	7	91–222	86–632
*TaHSP90B-7A*	*TraesCS7A01G529900*	15	7	19–59	67–232
*TaHSP90B-7B*	*TraesCS7B01G446900*	15	13	24–66	65–203
*TaHSP90B-7D*	*TraesCS7D01G517800*	15	7	20–78	65–235
*TaHSP90C1-5A*	*TraesCS5A01G251000*	19	2	32–89	29–190
*TaHSP90C1-5B*	*TraesCS5B01G249000*	19	4	22–119	77–422
*TaHSP90C1-5D*	*TraesCS5D01G258900*	19	2	15–99	27–220
*TaHSP90C2-5A*	*TraesCS5A01G101900*	20	5	5–12	4–24
*TaHSP90C2-5B*	*TraesCS5B01G106300*	20	9	6–12	19–104
*TaHSP90C2-5D*	*TraesCS5D01G113700*	20	7	7–13	11–56

Sequence analysis showed that the protein sequence identities of the three *TaHSP90* homeologs were above 96% (Supplementary Table 1). Accordingly, the protein sequence motifs (Supplementary Figure 2A and Supplementary Table 2) and gene structures (Supplementary Figure 2B) were also highly consistent among three TaHSP90 homeologs. Particularly, all the three *TaHSP90* homeologs contained the same exon number, and that is 3, 3, 15, 19, and 20 for subfamily AA, AB, B, C1, and C2, respectively. These results showed that the sequences and gene structures are highly conserved within three *TaHSP90s* homeologs in hexaploid wheat, which were consistent with the reports in *Populus* and *Brachypodium distachyon* (Zhang et al., 2013, 2017).

### Conserved Heat Response Pattern Among Three *TaHSP90* Homeologs

**FIGURE 3:**
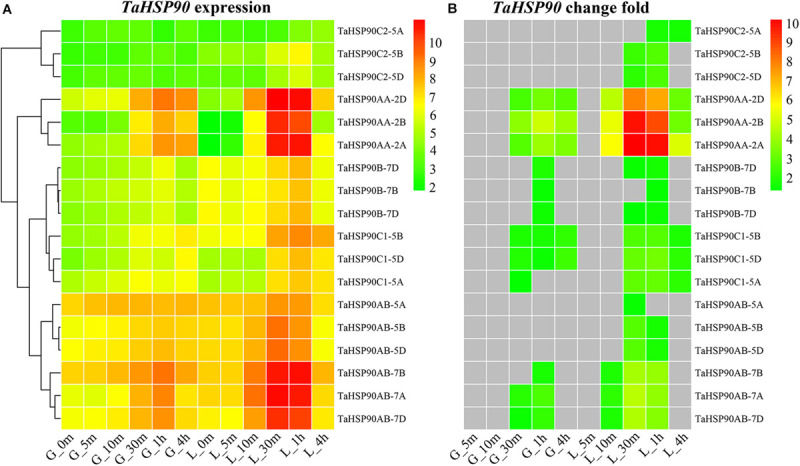
Expression and response patterns of *TaHSP90s* at different heat stress treatment (37°C) time points. The samples were indicated by a character that refers to the organ, followed by the heat stress time point (for example, “G_5m” indicated that grains were treated with heat stress for 5 m). G, grains; L, flag leaves; m, minute; h, hour. **(A)** Expression level of each *TaHSP90* in 12 samples. The heatmap was drawn by the “pheatmap” package in R software based on log2-transformed (FPKM + 1) value. Complete linkage clustering analysis was performed with maximum distance. **(B)** Change fold of each *TaHSP90* under heat stress. Change fold was calculated by comparing the expression abundance in each heat stress condition to that in normal condition (G- or L-0m). Log2-transformed change fold was shown.

Using the dynamic and intensive heat response transcriptomes of filling grains and flag leaves of wheat generated by our previous study (Wang et al., 2019), we investigated the heat response patterns of *TaHSP90s* and found that the heat stress response trend of each member of three *TaHSP90* homeologs was largely conserved, although the expression abundance was slightly different (Figure 3A and Supplementary Figure 3). For example, all the three *TaHSP90AAs* homeologs were lowly expressed under normal conditions in grains and flag leaves, and they were sharply upregulated (fold change ≥ 2 and FDR-adjusted *P-value* < 0.01) at 10 and 30 min heat stress treatment point in flag leaves, and grains, respectively (Figure 3B), consistent with *HSP90AAs* that were highly heat inducible (Swindell et al., 2007; Hu et al., 2009). Besides, all of the 18 *TaHSP90s* were heat responsive in flag leaves; however, it is also worth noting that all *TaHSP90C2s* (*TaHSP90C2-5A*, *TaHSP90C2-5B*, and *TaHSP90C2-5D*) and three *TaHSP90ABs* homeologs (*TaHSP90AB-5A*, *TaHSP90AB-5B*, and *TaHSP90AB-5D*) did not respond to heat stress in grains, suggesting a distinct response network between these two organs. In conclusion, together with the high level of sequence and gene structure conservation, these results demonstrated that no significant divergence of the three *TaHSP90* homeologs occurred at transcriptional level in hexaploid wheat, though the heat response patterns of *TaHSP90s* were highly dynamic between different heat durations, gene subfamilies, and organs.

### Large Number of Novel Isoforms Generated by TaHSP90s Under Heat Stress

Recent findings have suggested the importance of AS regulation in abiotic stress response (Reddy et al., 2013; Jiang et al., 2017; Keller et al., 2017; Laloum et al., 2018; Liu et al., 2018). Using the qualitative and quantitative heat response transcriptomes of filling grains and flag leaves produced by combining second- and third-generation sequencing in our previous study (Wang et al., 2019), we comprehensively investigated the roles of AS in the heat response of *TaHSP90s*.

First, a total of 126 isoforms of *TaHSP90s* were identified from our data, including the 36 isoforms that had been annotated in IWGSC RefSeq v1.0 and 90 newly identified isoforms (Supplementary Tables 3, 4 and Supplementary Figures 4, 5). The number of isoforms per *TaHSP90* gene ranged from 2 to 13, with an average of 9.0, 8.5, 7.0, 6.3, and 2.7 for the subfamily B, AB, C2, AA, and C1, respectively (Table 1). Although the exon–intron structures were highly conserved among the three *TaHSP90* homeologs (Supplementary Figure 2B), the isoform numbers generated by the three *TaHSP90* homeologs were such distinct. The most distinct change was observed in the three *TaHSP90AB* homeologs on chromosome group 7; the isoform numbers ranged from 6 to 13. In this case, the highly conserved *TaHSP90s* homeologs would possibly diverge at AS level by generating different isoform numbers under heat stress.

Second, with the quantified information of each isoform, we found that the six *TaHSP90s* (*TaHSP90AB-5A*, *TaHSP90AB-5B*, *TaHSP90AB-5D*, *TaHSP90C2-5A*, *TaHSP90C2-5B*, and *TaHSP90C2-5D*) that were not heat responsive in grains generated some isoforms that responded to heat stress with transcriptional regulation (fold change ≥ 2 and FDR-adjusted *P-value* < 0.01) (Supplementary Figure 6). Thus, these transcriptionally heat-responsive isoforms extended our understanding of the transcriptional regulation of *TaHSP90s* and further revealed the complexity of the heat stress response for this gene family.

**FIGURE 4:**
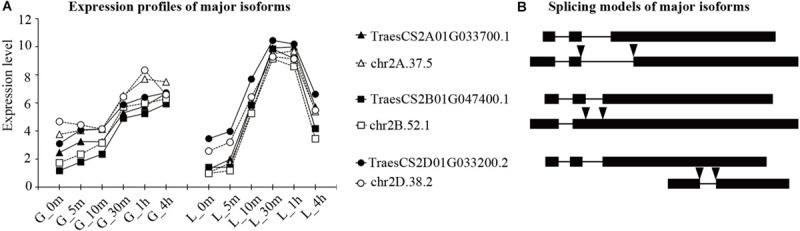
Expression profiles and splicing modes of major isoforms generated by three *TaHSP90AA* homeologs. Isoform names are shown for both **(A)** and **(B)**. **(A)** Expression profiles of major isoforms of *TaHSP90AAs*. Expression level is shown as the log2-transformed (FPKM + 1) value. The samples are indicated by a character that refers to the organ, followed by the heat stress duration (for example, “G_5m” indicated that grains were treated with heat stress for 5 m). G, grains; L, flag leaves; m, minute; h, hour. **(B)** Splicing modes of the major isoforms of *TaHSP90AAs*. Solid boxes represented exons, lines represented introns, and arrows indicated the splicing sites that differed from the longest intact coding sequence.

Next, to characterize the predominant isoforms of each *TaHSP90* gene that may play more important roles, we introduced the IEP, which was calculated as the expression abundance ratio of one isoform to all isoforms generated by the same gene. An isoform with an average IEP of more than 30% across all of the time points in an organ was regarded as a major isoform, an isoform with an IEP less than 5% in all time points was regarded as a rare isoform, and all other isoforms were classified as minor isoforms. This analysis led to the classification of isoforms into major (30), minor (44), and rare (52) isoforms (Supplementary Table 5). For 18 *TaHSP90s*, one *TaHSP90* (*TaHSP90AB-5B*) generated three major isoforms, 12 *TaHSP90s* generated two major isoforms, and five *TaHSP90s* generated one major isoform. Interestingly, among the two or three major isoforms, one was already annotated in IWGSC RefSeq v1.0 and contained the longest complete coding region, which potentially encoded a functional peptide containing both the HATPase domain and HSP90 domain. However, another major isoform was newly discovered from our hybrid sequencing data, and this possessed only the HSP90 domain that potentially encoded truncated peptides. Furthermore, expression analysis showed that the major isoforms generated by the same gene had comparable expression levels and response patterns (Figure 4 and Supplementary Figures 7, 8), making it intriguing as to what roles these newly discovered isoforms played in the heat stress response.

### Varied Number and Splicing Modes of Major Isoforms Generated by TaHSP90 Homeologs

The above transcription analysis showed that the expression abundance and response patterns of three *TaHSP90* homeologs were conserved. However, regarding the number of major isoforms generated by three *TaHSP90* homeologs, only one or two homeologs generated two major isoforms in subfamily AB, B, C1, and C2, with the exception of subfamily AA in which each *TaHSP90* homeolog generated two major isoforms (Figure 4 and Supplementary Figures 7, 8), demonstrating a varied major isoform number among three *TaHSP90* homeologs.

Furthermore, the newly identified major isoforms (NIMIs) from our hybrid sequencing data also exhibited different exon and intron compositions among the three *TaHSP90* homeologs, although they had the same gene structure, implying that the three *TaHSP90* homeologs may exploit different peptides to respond to heat stress. For example, among the three *TaHSP90AAs* homeologs, the NIMI of *TaHSP90AA-2A* underwent AS at both the 5′ (Alt5’SS) and the 3′ ends of the first intron (Alt3’SS). The NIMI of *TaHSP90AA-2B* underwent intron retention. The NIMI of *TaHSP90AA-2D* underwent exon skipping (Figure 4). In conclusion, the number and isoform structure generated by the three *TaHSP90* homeologs were significantly different, despite the three *TaHSP90* homeologs being conserved at the sequence level and at the transcriptional response level. The differential number and isoform structure possibly suggested a new direction for evolutionary divergence of *TaHSP90* homeologs in hexaploid wheat.

### Differential AS Responses of Three TaHSP90 Homeologs

**FIGURE 5:**
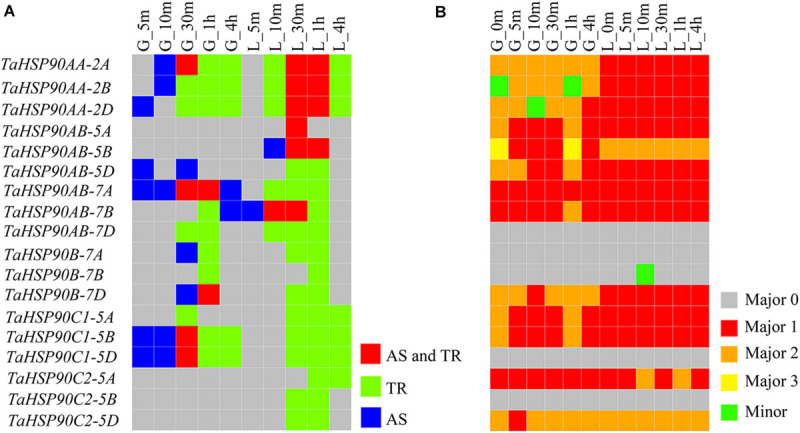
Differential AS response of *TaHSP90s* under heat stress. The samples are indicated by a character that refers to the organ, followed by the heat stress duration (for example, “G_5m” indicated that grains were treated with heat stress for 5 m). G, grains; L, flag leaves; m, minute; h, hour. **(A)** Responses of *TaHSP90s* at different levels under different conditions. AS, alternative splicing response. TR, transcriptional response. **(B)** The most abundant isoform of each *TaHSP90* in each sample. For the five *TaHSP90s* (*TaHSP90AB-7D*, *TaHSP90B-7A*, *TaHSP90B-7B*, *TaHSP90C1-5D*, *TaHSP90C2-5B*), they only generated one major isoform (Major 0). For the other *TaHSP90s*, which generated more than one major isoform; Major 1 represented the major isoform with the complete reading frame, and Major 2 and Major 3 (for *TaHSP90AB-5B* only) represented the other major isoforms. Minor represented the minor isoform.

Comparing the control and heat stress treatment samples, we identified 12 *TaHSP90s*, which responded to heat stress by generating new isoforms or changing the expression level of highly expressed isoforms with the criterion defined in our previous study (Figure 5A) (Wang et al., 2019). Interestingly, some *TaHSP90s* that did not respond to heat stress with transcriptional regulation did respond to heat stress with AS regulation, particularly in grains under short time (5 and 10 min) heat stress, extending our understanding of the heat response of *TaHSP90s*. Significantly, for the three *TaHSP90* homeologs in subfamily B, C1, and AB on chromosome group 7, only one or two homeologs responded to heat stress with AS regulation, and in subfamily AA, though all three *TaHSP90AAs* underwent AS responses, but the AS responses occurred at different heat stress time points in grains. These results demonstrated the differential responses of three *TaHSP90s* homeologs at the AS regulation level.

Furthermore, using the qualitative and quantitative isoforms, we investigated the isoform with the highest abundance for each *TaHSP90* at each heat stress time point sample (Figure 5B). The highest abundance isoforms generated by three *TaHSP90* homeologs at specific time point were also distinct, providing another evidence for the differential AS responses and suggesting possible diverged evolution. In conclusion, inconsistent with the conserved sequences and transcriptional regulation, AS response diverged among the three *TaHSP90* homeologs, extending our understanding about the functional conservation and divergence of this gene family in hexaploid wheat.

## Discussion

HSP90s play vital roles in plant growth and stress response (Wang et al., 2004; Wang et al., 2016; Gil et al., 2017). In this study, we first performed a genome-wide analysis of *HSP90s* in hexaploid wheat and its progenitors. The copy numbers of *HSP90s* among these species were not consistent with polyploidy level. Next, we comprehensively analyzed the heat response patterns of *TaHSP90s* and found that AS diversified the heat response of *TaHSP90s*, suggesting different options for functional studies and breeding strategies. Meanwhile, our results provided a new perspective for understanding about evolutionary conservation and divergence for the homeologous genes in polyploidy species.

It has been reported that *HSP90AAs* were dramatically upregulated during heat stress, and *HSP90ABs* were constitutively expressed in Arabidopsis (Swindell et al., 2007) and rice (Hu et al., 2009). However, with more intensive time course transcriptomes and different organs, we showed that all of the *TaHSP90s* were heat responsive under at least one time point in flag leaves, suggesting that this was a specific feature in hexaploid wheat or hinting that more investigations should be performed in Arabidopsis and rice to draw a conclusion. However, it was noteworthy that our data mainly focused on the short time heat response, and the *TaHSP90s* that do not respond to heat stress in grains may also respond to heat stress in other conditions.

The differentiation and subfunctionalization of homeologous genes are intensified by stress and are thought to contribute to the acclimation of polyploidy plants to stress (Dong and Adams, 2011; Liu et al., 2015; Powell et al., 2017). For example, about 68% of homeologous genes display expression partitioning according to the extent of stress responsiveness in hexaploid wheat (Liu et al., 2015). The homeolog-specific expression patterns of homeologous genes were also widely reported in wheat genes, resulting in different morphological phenotypes like lateral root number (Wang et al., 2018a), root hair length (Han et al., 2016), and other domestication traits (Zhang et al., 2011). For HSP90s, members of three *TaHSP90C2* homeologs and three *TaHSP90AB* homeologs were also predicted to experience expression partitioning under drought stress and combined drought-heat stress, but not under only heat stress (Liu et al., 2015). Similarly, in our analysis, the heat expression trends and fold changes of *TaHSP90* members were not significantly distinguishable between each other in three *TaHSP90* homeologs. Thus, it seemed like the three *TaHSP90* homeologs were conserved and had not undergone subfunctionalization or neofunctionalization in heat response at transcriptional level, which was consistent with the highly conserved sequences and motifs.

However, in our subsequent analysis, we found that the numbers of major isoforms were distinct among three *TaHSP90* homeologs, and further investigations revealed that the AS modes of the major isoforms generated by the three *TaHSP90* homeologs were also not conserved, suggesting that the differentiation of *TaHSP90* homeologs may occur at the AS level. Different splicing patterns have also been characterized among homeologous genes in allopolyploid cotton (Wang et al., 2018b). More than 51% of the homeologous genes generated isoforms containing different structure in allotetraploid cottons (Wang et al., 2018b). Theoretically, the differentiation in AS that resulted in distinct transcripts may lead to the diversified functions of homeologous genes (Long et al., 2013). Thus, divergent AS patterns and differential AS responses may contribute to the functional divergence and differential evolution of *TaHSP90* homeologs, changing our understanding of the conservation of *HSP90s* in terms of expression profile and function.

In this study, *TaHSP90s* were found to generate many novel isoforms in the grain-filling stage under heat stress. Contrary to that, the expression of the abnormal isoforms was generally lower than those of the full-length isoforms of *Lipoxygenase* members in the tea plant in response to low temperature (Zhu et al., 2018), the expression levels of the truncated major isoforms and their full-length counterparts were found to be comparable in the current study. It was worthy to note that the peptides encoded by the truncated major isoforms only contain the HSP90 domain (Supplementary Table 3), making the roles of these truncated major isoforms intriguing. It is well known that the HATPase domain is responsible to bind ATP; the HSP90 domain is responsible for homodimerization and binding to clients (Schopf et al., 2017). We proposed that the truncated peptides modify their original functions by modulating the domain composition. For example, the novel major isoform of *TaHSP90AA-2A* possibly encoded a peptide containing the intact HSP90 domain but lost the HATPase domain. The truncated peptide may still form a homodimer but fail to bind clients without the ability to bind ATP; it seemed to decrease the protecting capacity of HSP90, and this was quite different to the AS regulation of *HSFA2* under heat stress (Sugio et al., 2009; Liu et al., 2013; Cheng et al., 2015). In contrast, a total of 70 isoforms, including 36 novel isoforms, were annotated to comprise both the HATPase and HSP90 domain. By this way, these isoforms would probably encode different proteins and significantly increase HSP90 protein diversity to protect different substrates. The contrary hypothesis of the roles of the different isoforms should be elucidated in further studies. Furthermore, as HSP90s were found to translocate into the nucleus under heat stress, alternative TaHSP90 isoforms finally were transported into the nucleus would result in a different expression of different heat-responsive genes. Another question was how many isoforms could be finally translated into proteins, as isoforms arose from AS always subsequently translated into normal or truncated proteins, or degraded by the non-sense-mediated decay pathway (Kalyna et al., 2012; Syed et al., 2012; Chaudhary et al., 2019)?

In grains, the *TaHSP90AAs*, the major heat-responsive *HSP90s*, tended to favor the novel truncated major isoform and minor isoform in most samples; the other eight *TaHSP90s* also favored different isoforms in different samples. These changes raised questions whether they correlated to the delay heat response in grains or thermotolerance of grains. These results also remind us that when investigating the functions of homeologous genes, expressions and splicing types of isoforms would be important and worthy of study.

About 40% of the differentially spliced genes were also found to be regulated at the transcriptional level, inferring the vital role of the cooperation of AS and transcriptional regulation in heat response in hexaploid wheat (Liu et al., 2018). In the present study, all of the 18 *TaHSP90s* were transcriptionally regulated, and 12 of these were also AS regulated. The higher cooperation ratio of AS and transcriptional regulation may contribute to fine modulation of *TaHSP90s*, to match its key roles in heat stress response.

## Data Availability Statement

The datasets presented in this study can be found in online repositories. The names of the repository/repositories and accession number(s) can be found below: https://www.ncbi.nlm.nih.gov/sra/SRP128236.

## Author Contributions

SX and XW designed the study. YL, PZ, AZ, LM, SX, and XW analyzed the data. YL, SX, and XW wrote the manuscript. All authors contributed to the article and approved the submitted version.

## Conflict of Interest

The authors declare that the research was conducted in the absence of any commercial or financial relationships that could be construed as a potential conflict of interest.

## Footnotes

1 https://urgi.versailles.inra.fr/

2 http://plants.ensembl.org/

3 ftp://ftpmips.helmholtz-muenchen.de/plants/

4 http://smart.embl-heidelberg.de/

5 https://itol.embl.de/
