# Supplementary figures and images for: Alternative Splicing Diversified the Heat Response and Evolutionary Strategy of Conserved Heat Shock Protein 90s in Hexaploid Wheat (Triticum aestivum L.)
Source: Front Genet. 2020 Nov 27;11:577897. doi: 10.3389/fgene.2020.577897 (PMC7729002; doi:10.3389/fgene.2020.577897)

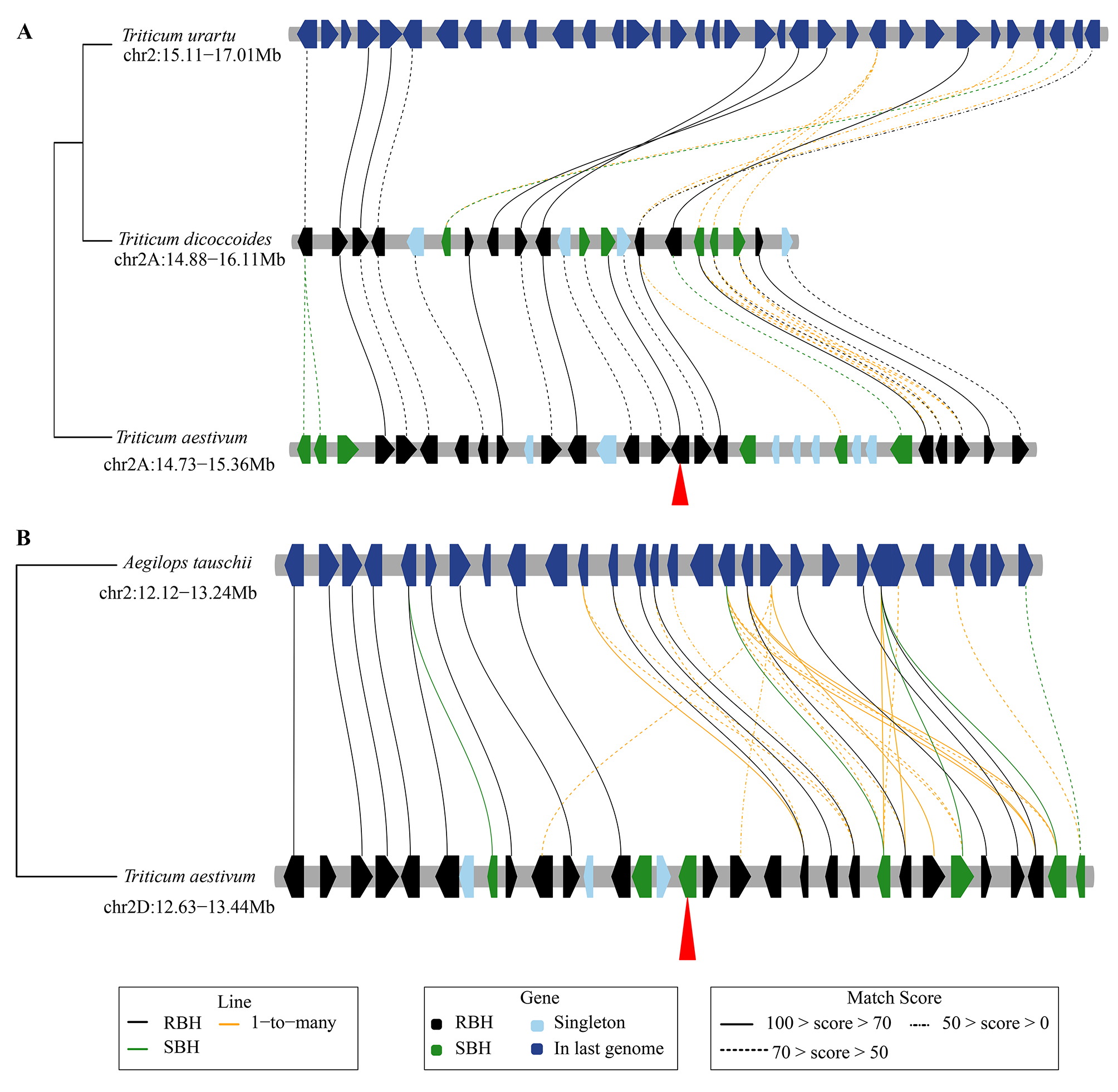

Supplement: Supplementary file 1 [file Image_1.TIF]

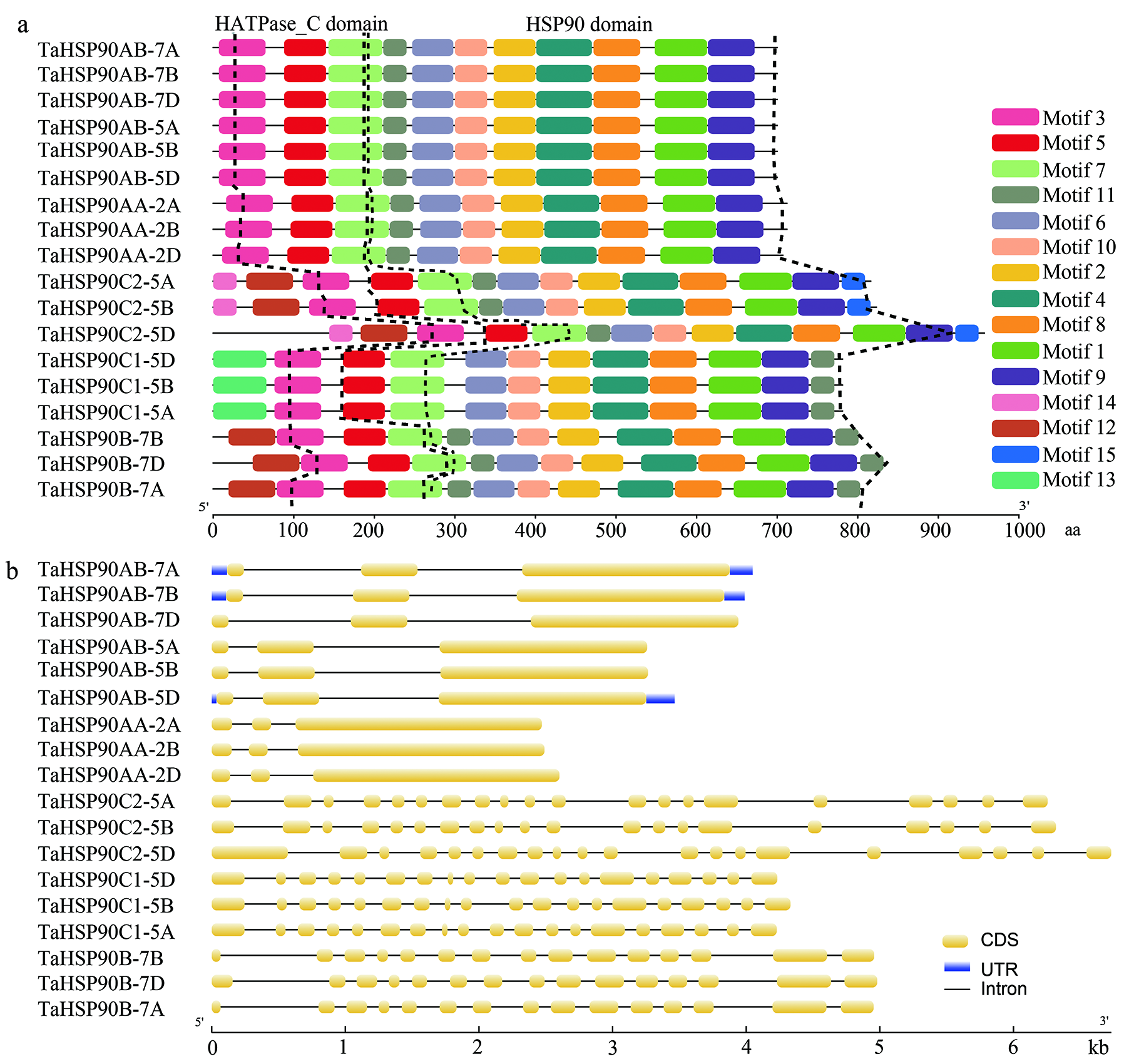

Supplement: Supplementary file 2 [file Image_2.TIF]

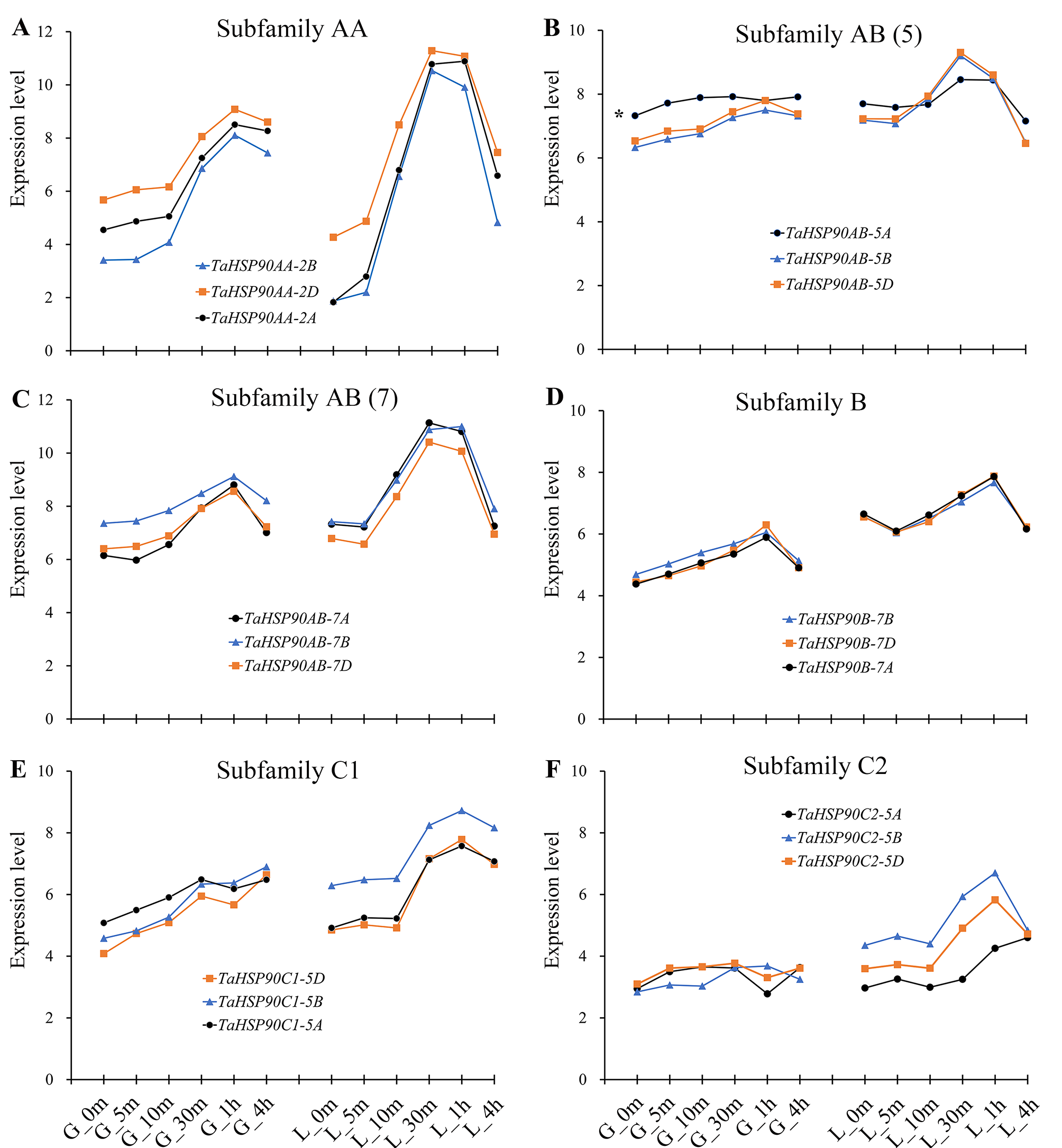

Supplement: Supplementary file 3 [file Image_3.TIF]

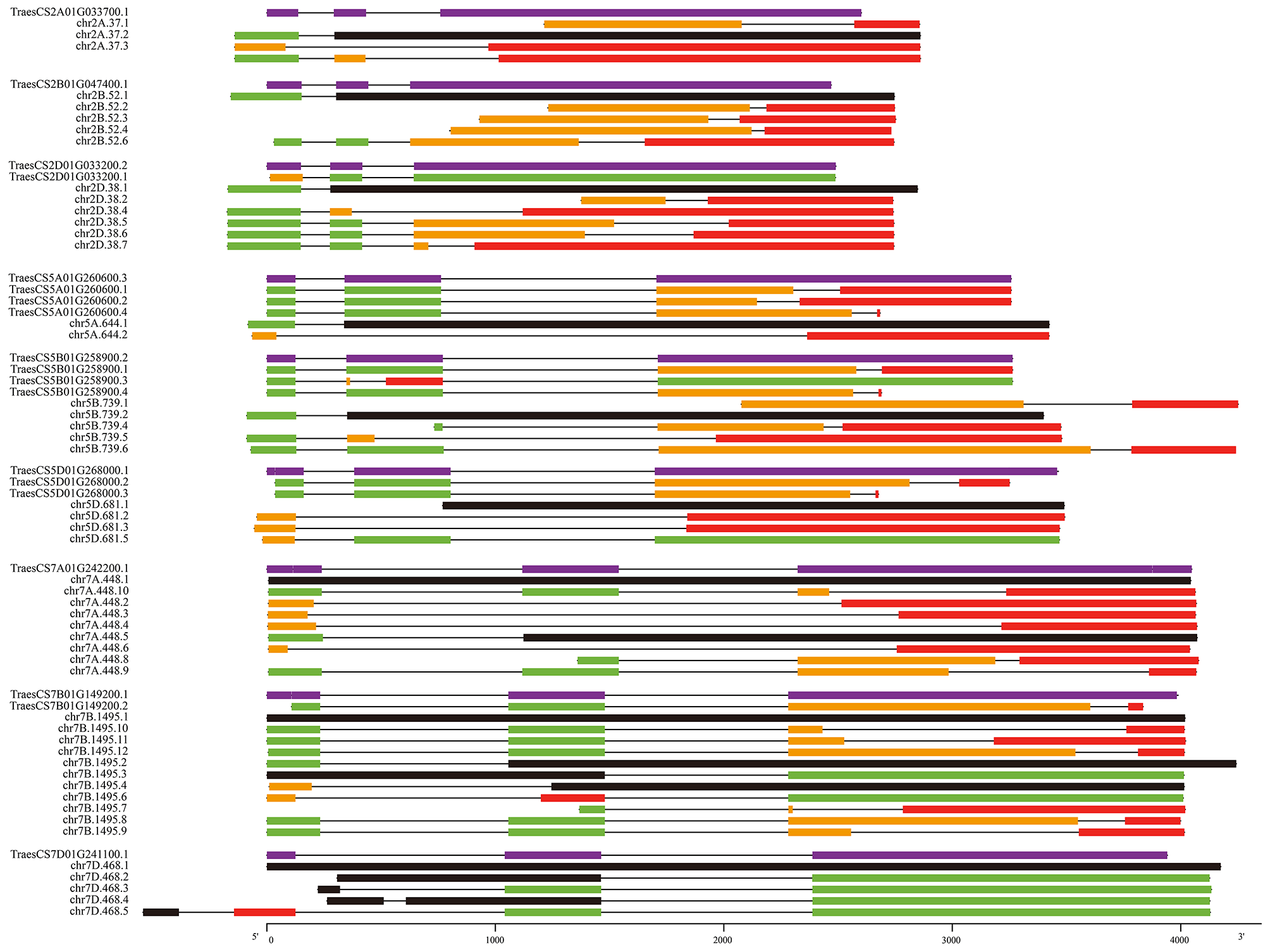

Supplement: Supplementary file 4 [file Image_4.TIF]

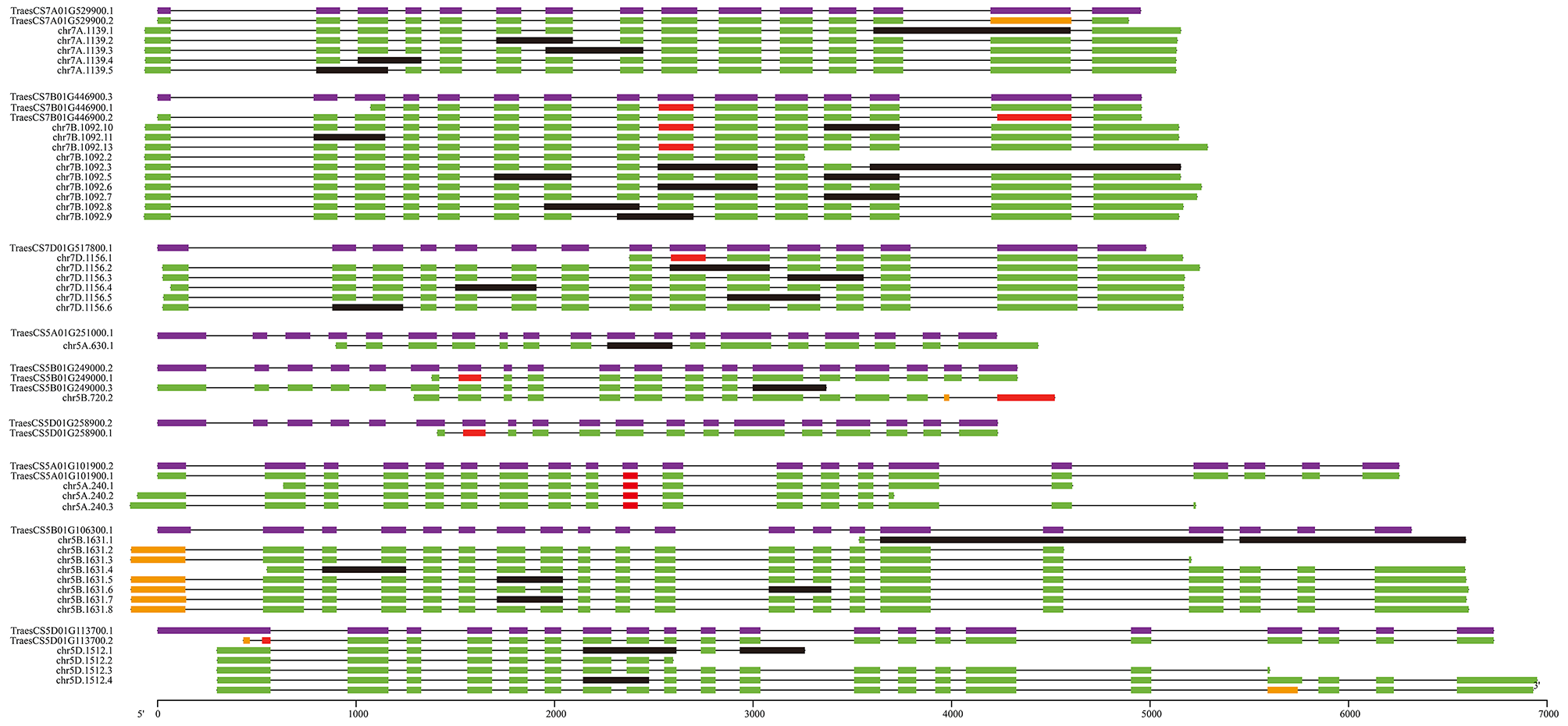

Supplement: Supplementary file 5 [file Image_5.TIF]

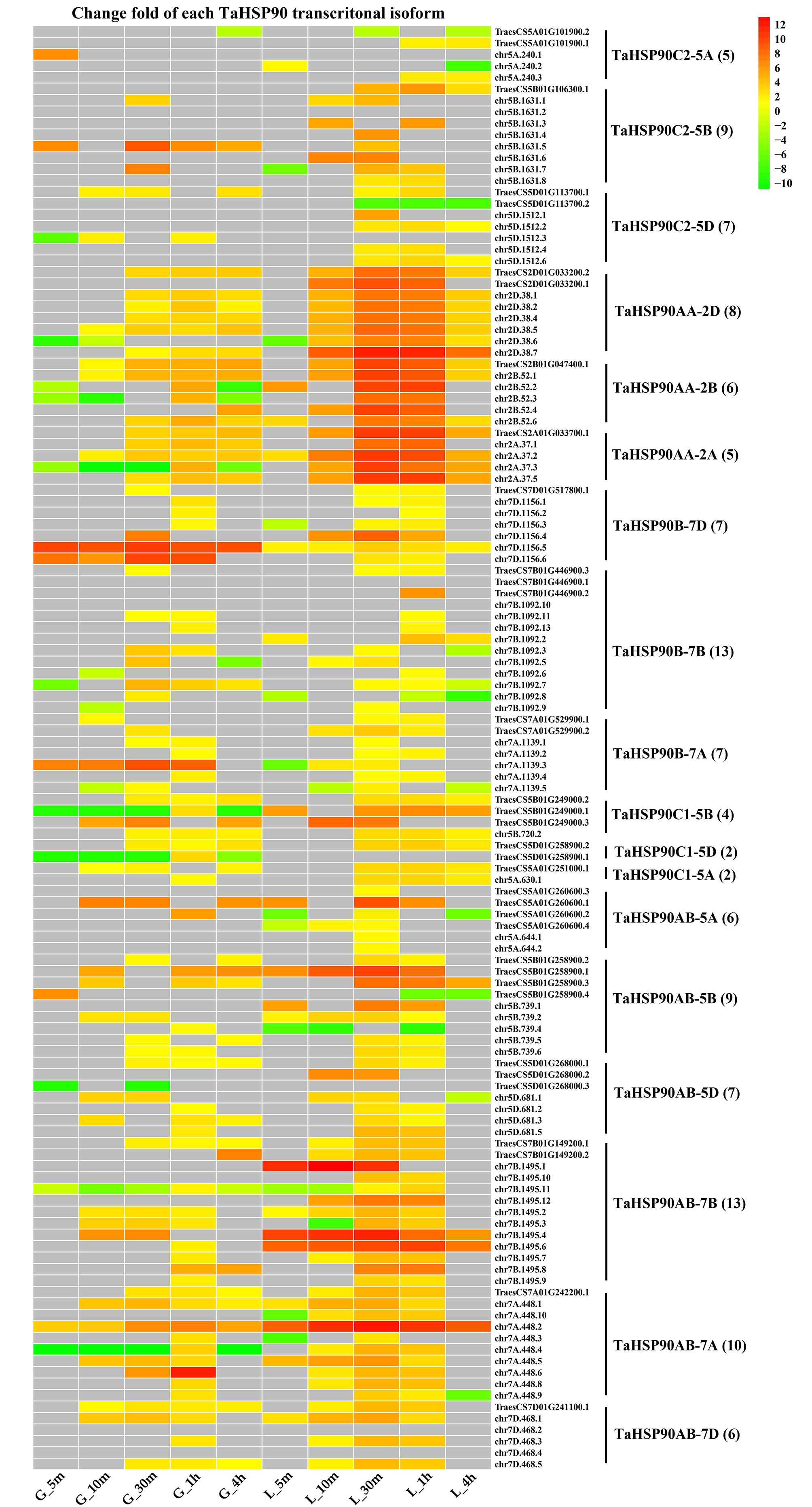

Supplement: Supplementary file 6 [file Image_6.TIF]

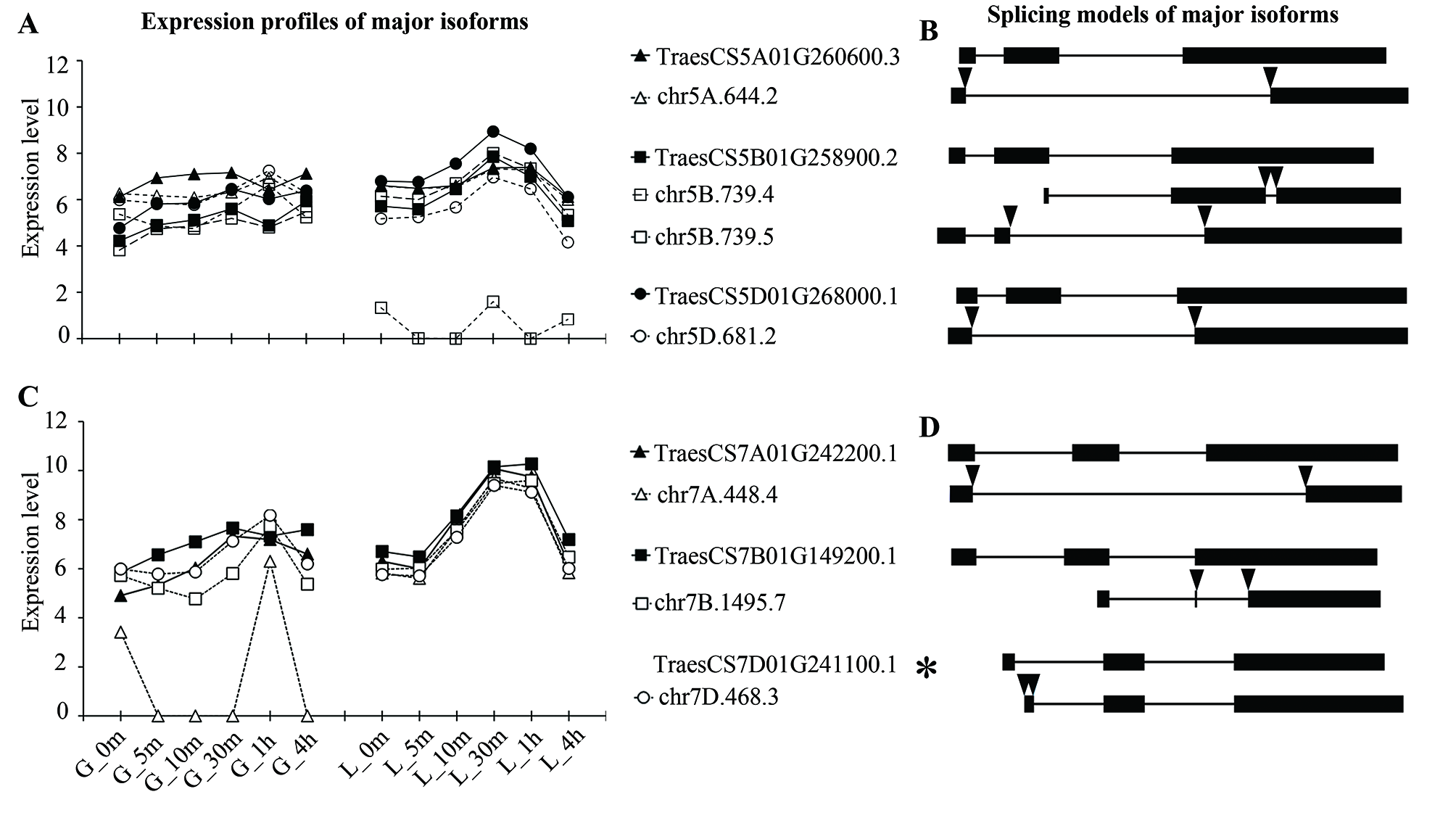

Supplement: Supplementary file 7 [file Image_7.TIF]

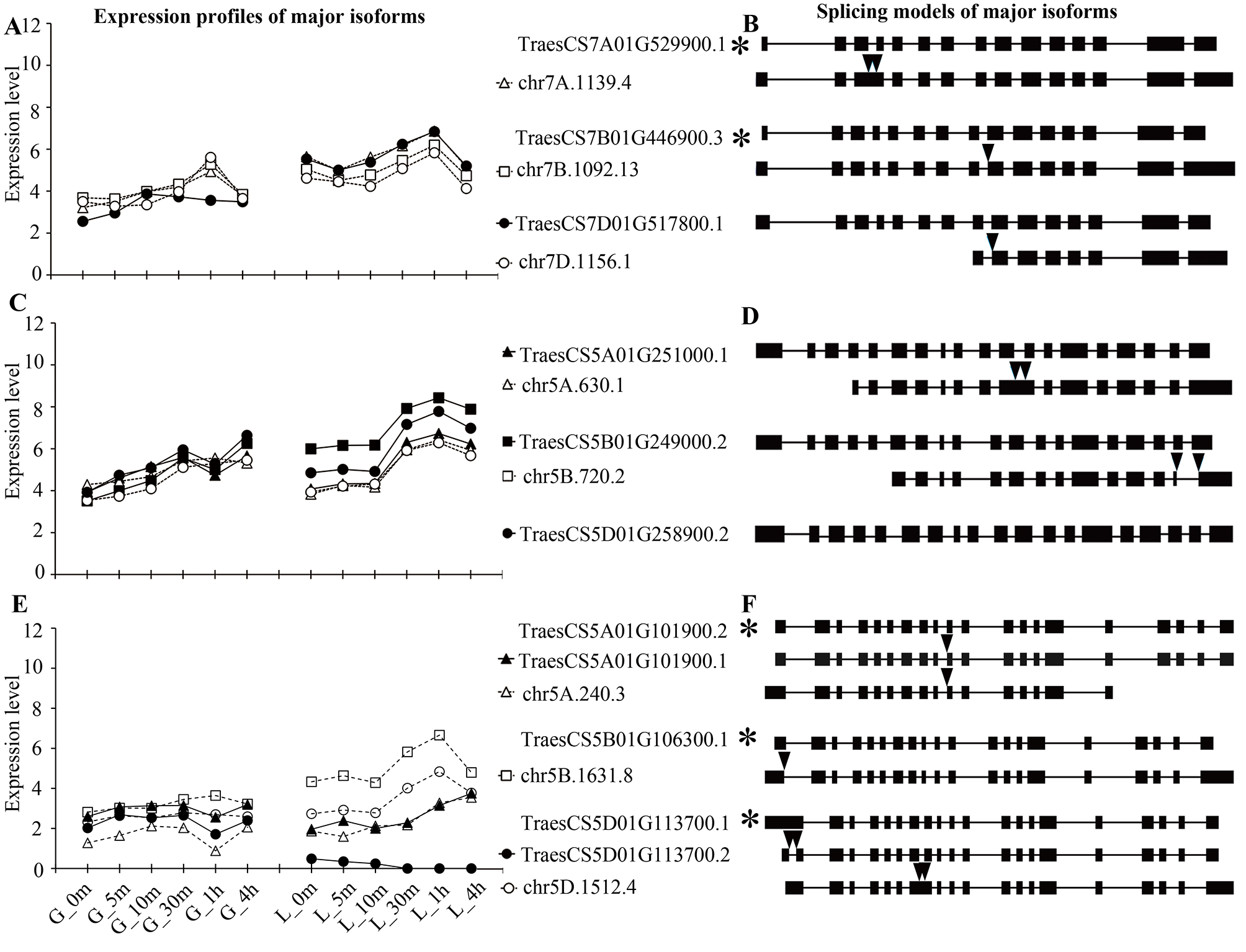

Supplement: Supplementary file 8 [file Image_8.TIF]
