# Supplementary material for: Alternative Splicing Diversified the Heat Response and Evolutionary Strategy of Conserved Heat Shock Protein 90s in Hexaploid Wheat (Triticum aestivum L.)
Source: Front Genet. 2020 Nov 27;11:577897. doi: 10.3389/fgene.2020.577897 (PMC7729002; doi:10.3389/fgene.2020.577897)
Supplement: Supplementary file 9 [file Data_Sheet_1.PDF]

Supplementary Material

1. Supplementary Tables

Supplemental Table 1. Sequence identities between HSP90 proteins
Supplemental Table 2. Detail information of MEME motifs
Supplemental Table 3. Information of TaHSP90 isoforms
Supplemental Table 4. GFF3 annotation file of TaHSP90 isoforms
Supplemental Table 5. Isoform classification based on FPKM value.

2. Supplementary Figures

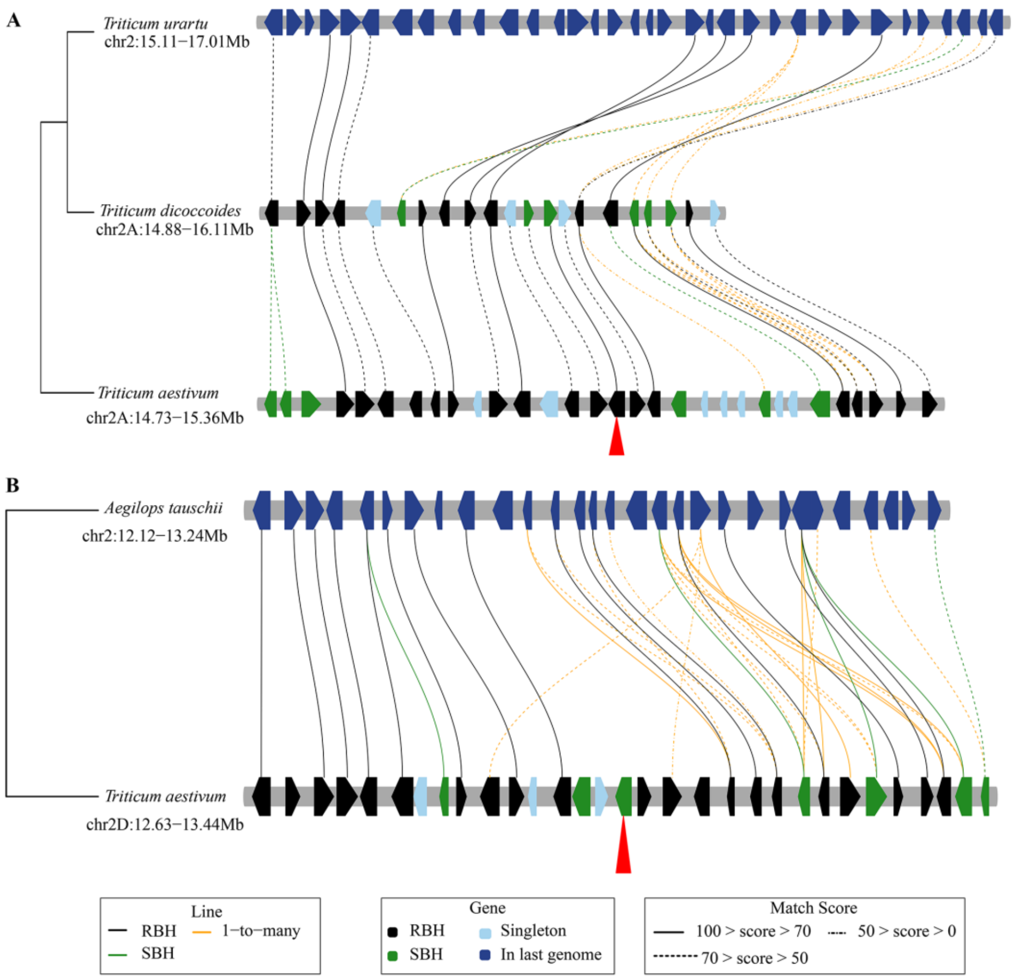

Supplementary Figure 1. Evolution of *TaHSP90AA-2A* (**A**), *TaHSP90AA-2D* (**B**) in diploid, tetraploid, and hexaploid species. Map was drawn on Triticeae-GeneTribe database (Chen et al. 2020). Red triangle referred to the *TaHSP90AA-2A* (**A**) and *TaHSP90AA-2D* (**B**) in hexaploid wheat. Genes were showed by pentagons, types of homoelogous relationship were indicated by different colors with different colors. RBH, reciprocal best hits. SBH, single-side best hits.

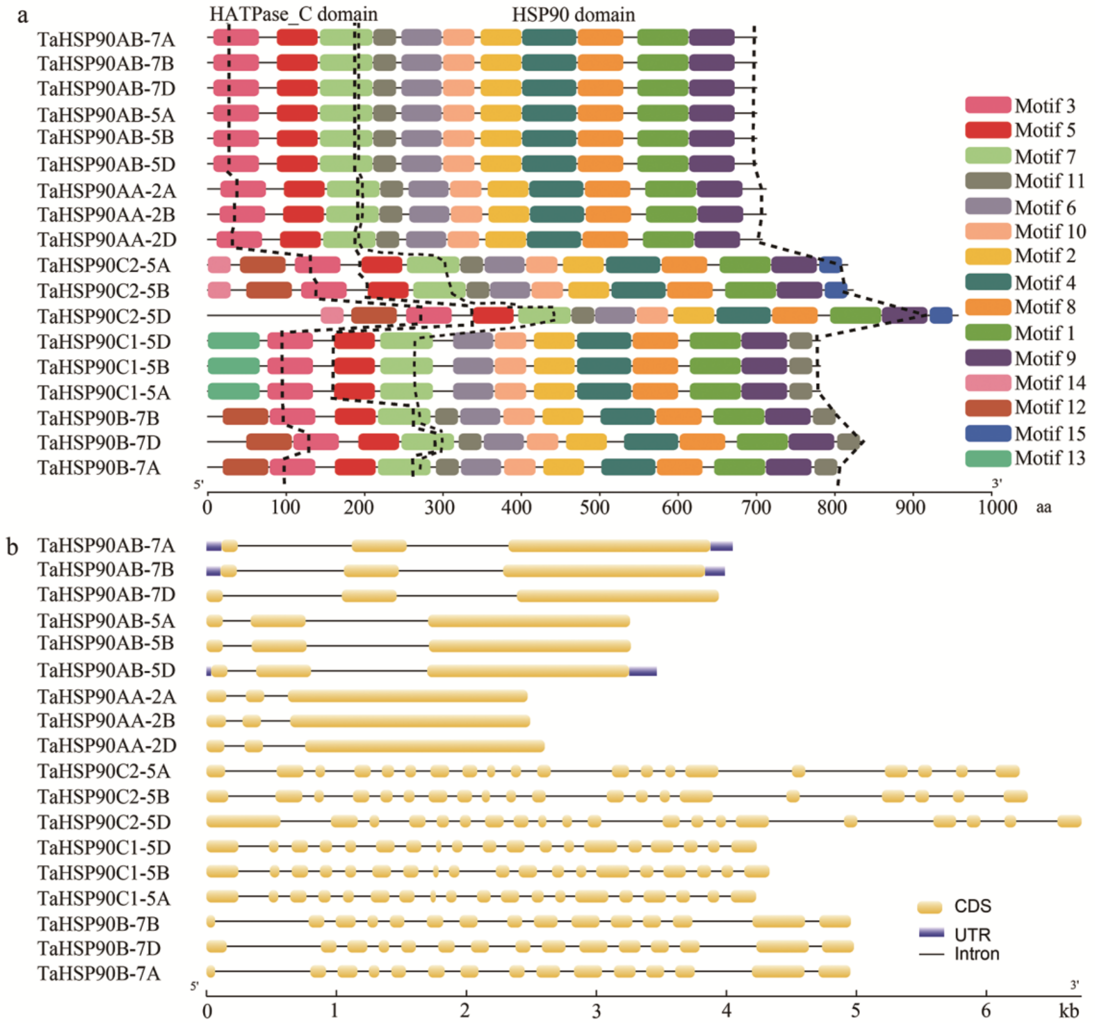

Supplementary Figure 2. **(A)** Protein motifs and **(B)** gene structures of *TaHSP90s*. Motif analysis was performed by MEME (http://meme-suite.org/). Gene structure analysis was carried out at Gene Structure Display Server 2.0 (http://gsds.cbi.pku.edu.cn/).

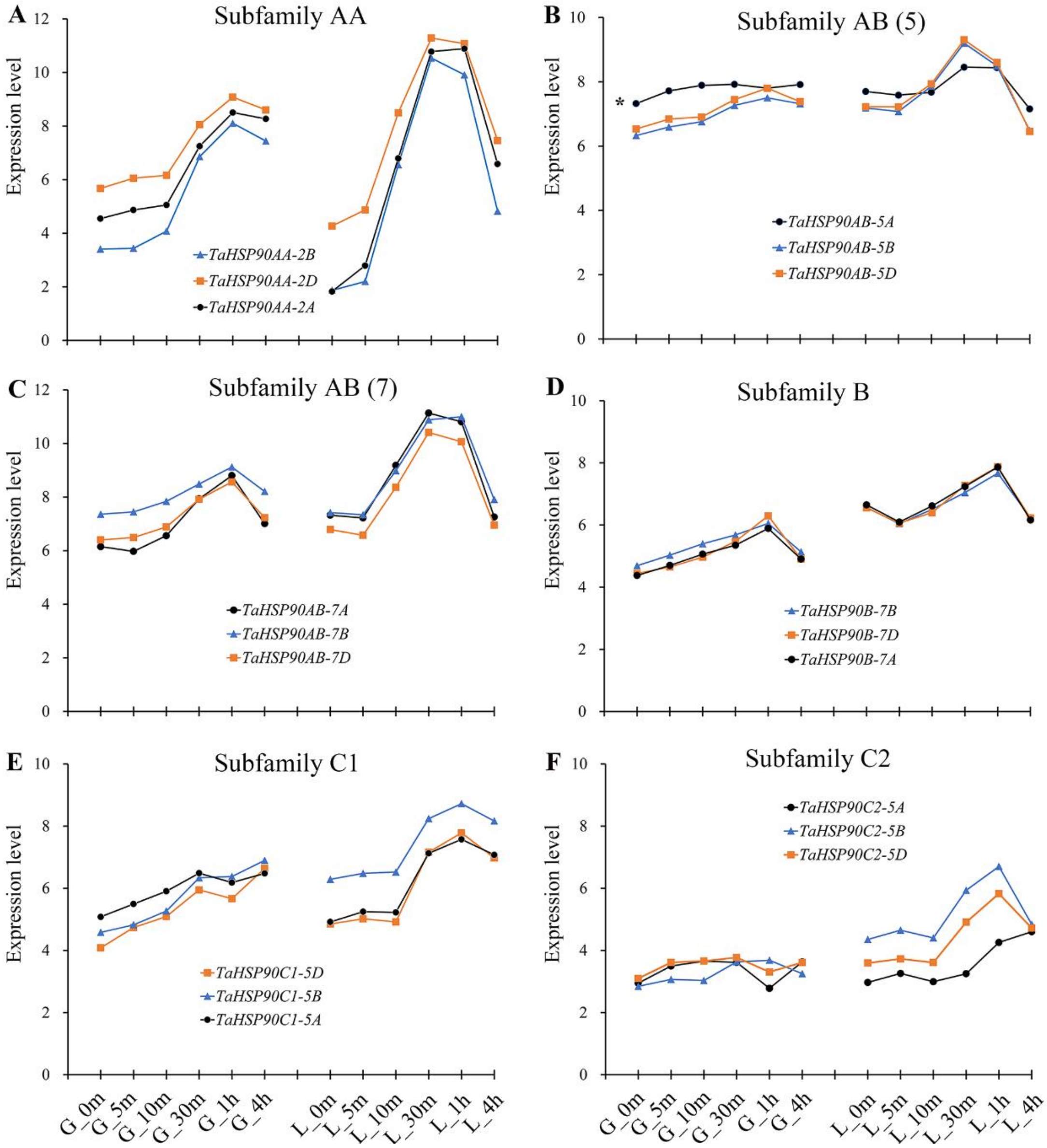

Supplementary Figure 3. Expression and response patterns of *TaHSP90s* at different heat stress treatment (37°C) time points. Expression level was showed as Log2-transformed (FPKM+1) value. The samples were indicated by a character that refers to the organ, followed by the heat stress time point (for example, 'G_5m' indicated that grains were treated with heat stress for 5 minutes). G, grains; L, flag leaves; m, minute; h, hour. G, grain. L, flag leaf. "0" is regarded as a control. The heat stress response patterns of three *TaHSP90* homeologs have no significant divergence at transcriptional level, with the exception of the comparison between *TaHSP90AB-5A* and *TaHSP90AB-5B* in which the response is significantly different at 0.01 level (two-tailed Student's *t* test).

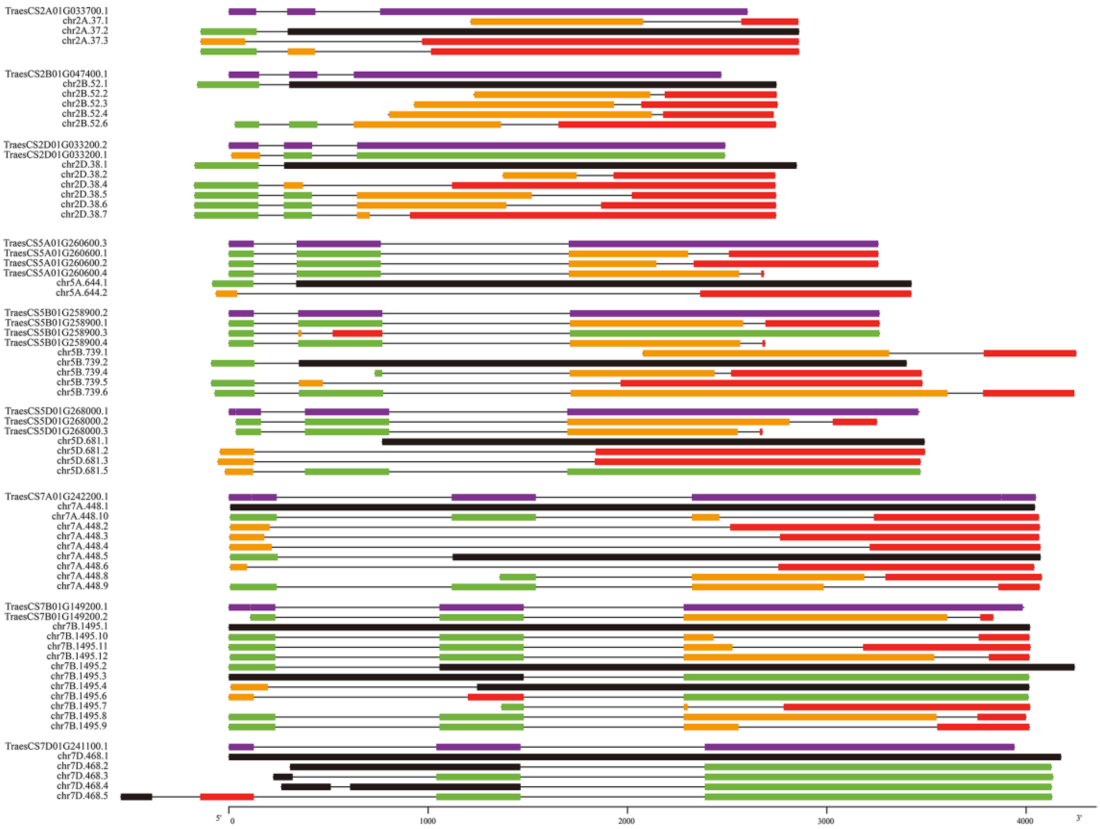

Supplementary Figure 4. Exon/intron structures of isoforms of *TaHSP90AAs* and *TaHSP90ABs*. Box represent exon, line represent intron. Purple boxes represent reference structures. Green box indicated the exon was same with reference exon. Black box indicated intron retention. Red box indicated alternative splicing site at 5' end of the exon. Orange box indicated alternative splicing site at 3' end of the exon. Differences in the 5' end of the first exon and 3' end of the last exon were not marked.

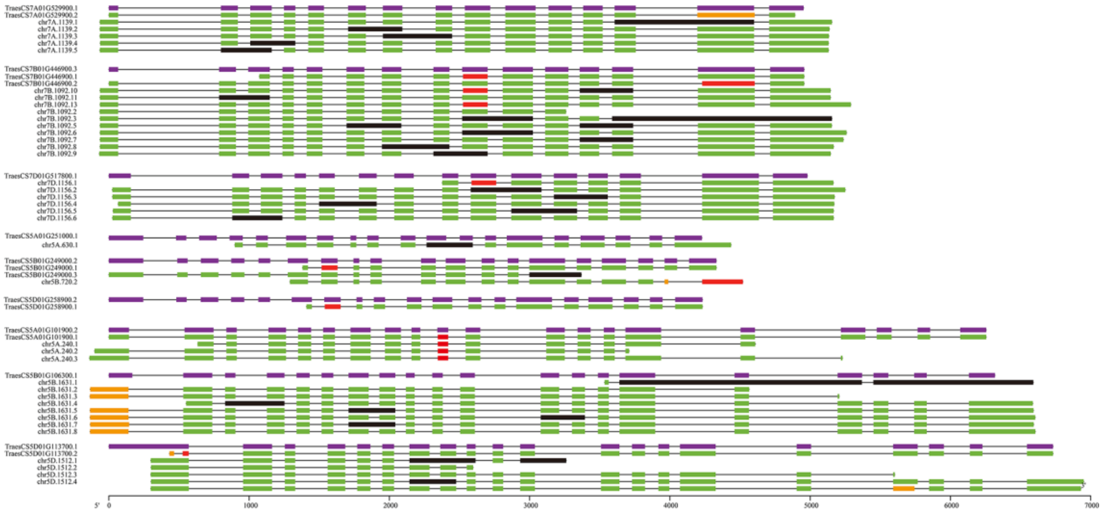

Supplementary Figure 5. Exon/intron structures of isoforms of *TaHSP90Bs* and *TaHSP90C1s* and *TaHSP90C2s*. Box represent exon, line represent intron. Purple boxes represent reference structures. Green box indicated the exon was same with reference exon. Black box indicated intron retention. Red box indicated alternative splicing site at 5' end of the exon. Orange box indicated alternative splicing site at 3' end of the exon. Differences in the 5' end of the first exon and 3' end of the last exon were not marked.

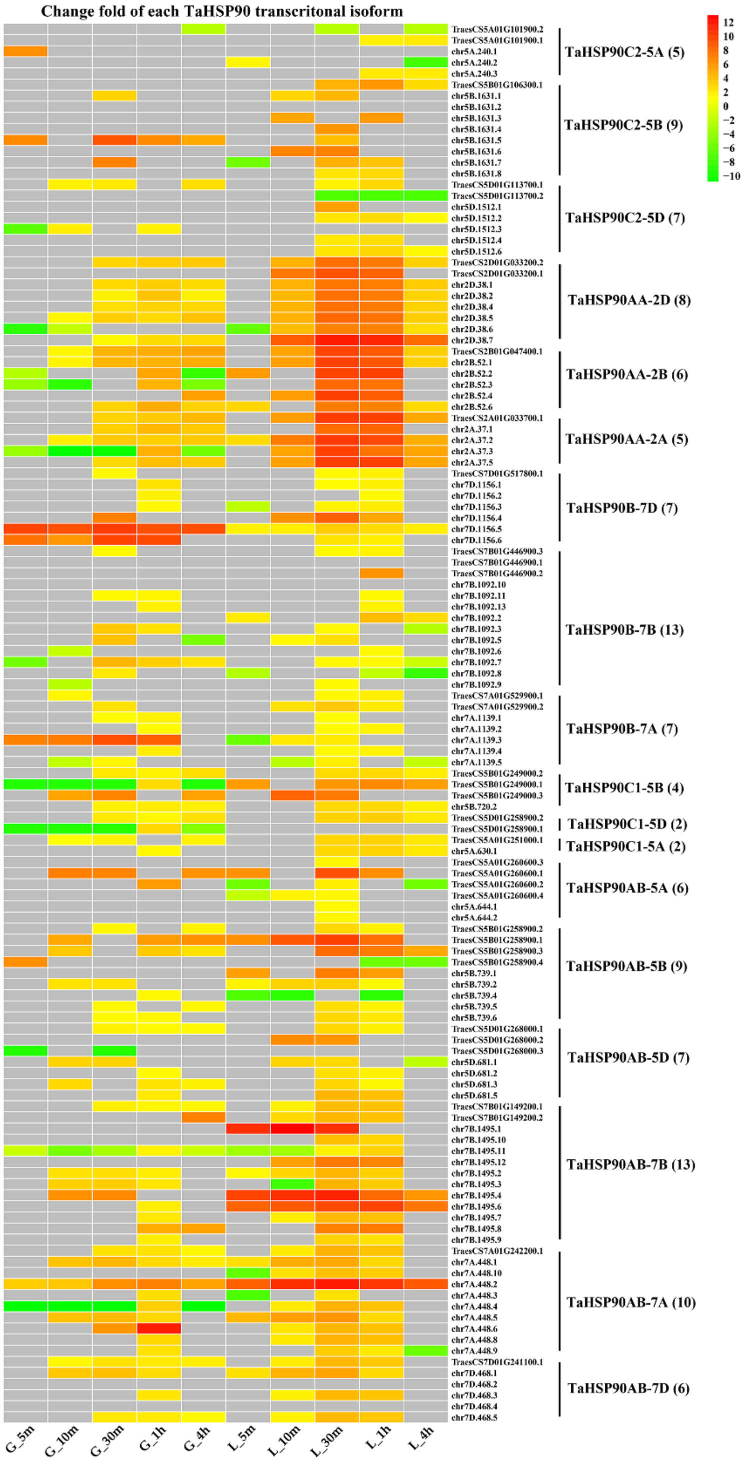

Figure 6. Change fold of each *TaHSP90* isoform at different heat stress treatment (37°C) time points. The samples were indicated by a character that refers to the organ, followed by the heat stress time points (for example, 'G_5m' indicated that grains were treated with heat stress for 5 minutes). G, grains; L, flag leaves; m, minute; h, hour. Log2-transformed change fold values were used to generate the heat map by 'pheatmap' package in R software. Isoforms generated by the same gene were indicated by the solid line next to the isoform names. For the isoform names, the names started with "Traes" represented those isoforms that were already annotated in IWGSC RefSeq v1.0, names started with "chr" represented those isoforms that were newly identified in our analysis. Isoform number of each gene was indicated in the brackets followed the gene name.

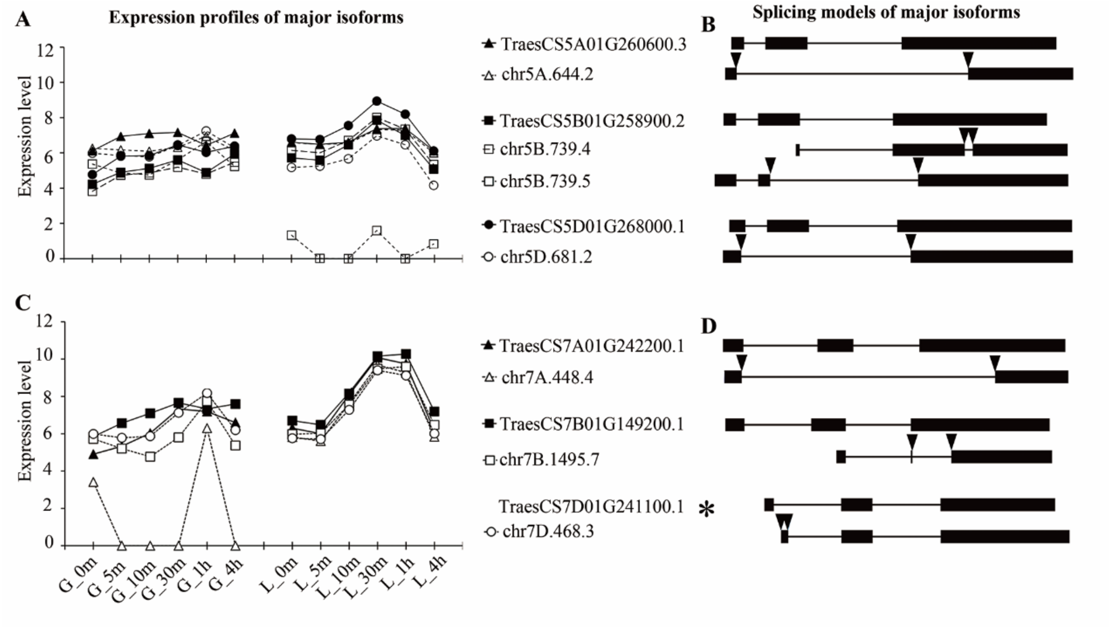

Supplementary Figure 7. Expression profiles and splicing modes of major isoforms generated by *TaHSP90AB* homeologs. Isoform names were showed for both left and right panels, and isoform name with asterisk indicated that was not a major isoform. Expression profiles of the major isoforms of *TaHSP90ABs* on chromosome group 5 **(A)** and group 7 **(C)**. Expression level was showed as the log2-transformed (FPKM+1) value. The samples were indicated by a character that refers to the organ, followed by the heat stress duration (for example, 'G_5m' indicated that grains were treated with heat stress for 5 minutes). G, grains; L, flag leaves; m, minute; h, hour. Splicing patterns of the major isoforms of *TaHSP90ABs* on chromosome group 5 **(B)** and group 7 **(D)**. Solid boxes represented exons, lines represented introns, arrows indicated the splicing sites that differed from the longest intact coding sequence.

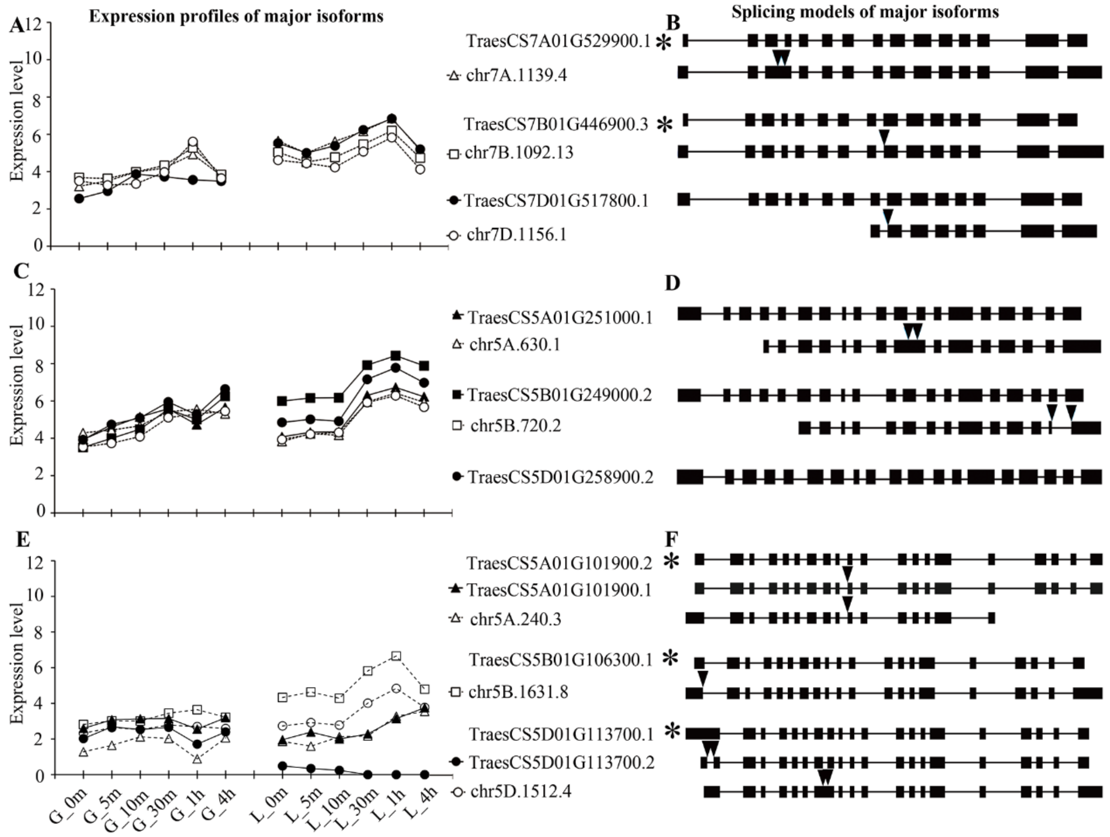

Supplementary Figure 8. Expression profiles and splicing modes of major isoforms generated by *TaHSP90B* homeologs, *TaHSP90C1* homeologs, and *TaHSP90C2* homeologs. Isoform names were showed for both left and right panels, and isoform name with asterisk indicated that was not a major isoform. Expression abundance of the major isoforms of *HSP90Bs* **(A)**, *HSP90C1s* **(C)**, and *HSP90C2s* **(E)**. Expression level was showed as the log2-transformed (FPKM+1) value. The samples were indicated by a character that refers to the organ, followed by the heat stress duration (for example, 'G_5m' indicated that grains were treated with heat stress for 5 minutes). G, grains; L, flag leaves; m, minute; h, hour. Splicing patterns of the major isoforms of HSP90Bs **(B)**, *HSP90C1s* **(D)**, and *HSP90C2s* **(F)**. Solid boxes represented exons, lines represented introns, arrows indicated the splicing sites that differed from the longest intact coding sequence.

Reference:

Chen, Y., Song, W., Xie, X., Wang, Z., Guan, P., Peng, H., et al. (2020) A collinearity-incorporating homology inference strategy for connecting emerging assemblies in Triticeae Tribe as a pilot practice in the plant Pangenomic era, Molecular Plant, doi:10.1016/j.molp.2020.09.019
